# Supplementary material for: Genome-wide association study for acute otitis media in children identifies FNDC1 as disease contributing gene
Source: Nat Commun. 2016 Sep 28;7:12792. doi: 10.1038/ncomms12792 (PMC5052699; doi:10.1038/ncomms12792)
Supplement: Supplementary Information — Supplementary Figures 1-12, Supplementary Tables 1-8 and Supplementary References [file ncomms12792-s1.pdf]

Supplementary Figures

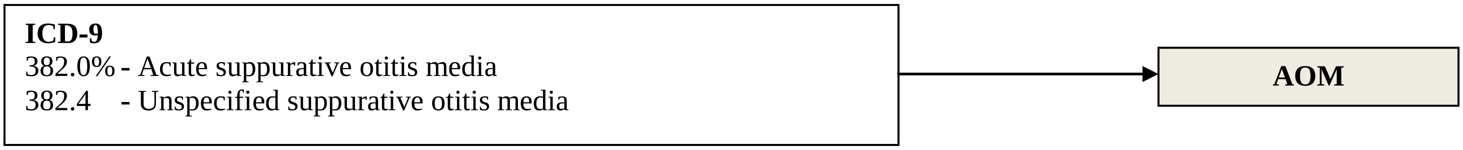

% is a wildcard character used in our database search, representing the inclusion of all subtypes of this ICD-9 code

Supplementary Figure 1: Flow chart of ICD-9 or procedure codes used as search term in CAG database (CHOP) to define AOM phenotype

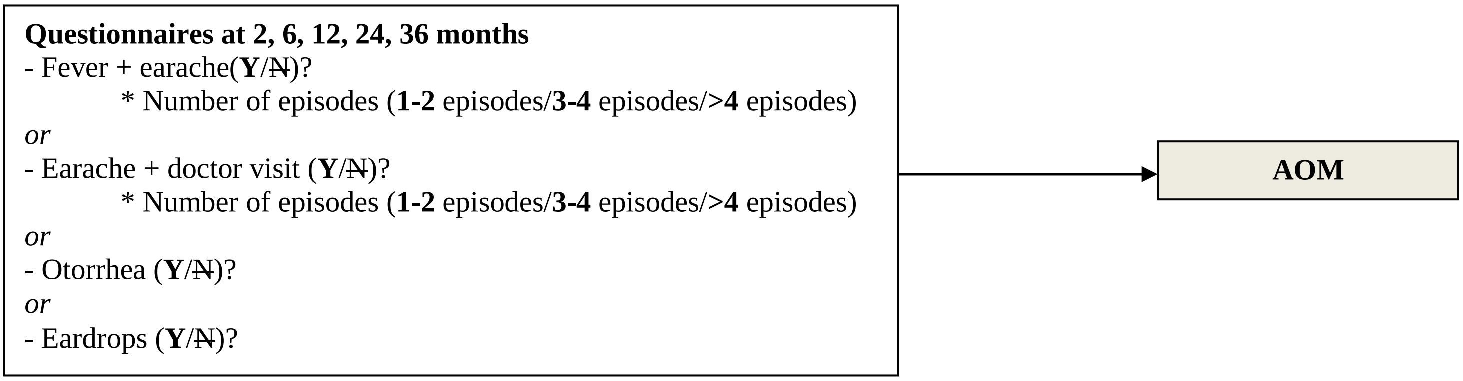

Supplementary Figure 2: Flow chart of questionnaire data used to define AOM phenotype at the Generation R Study.

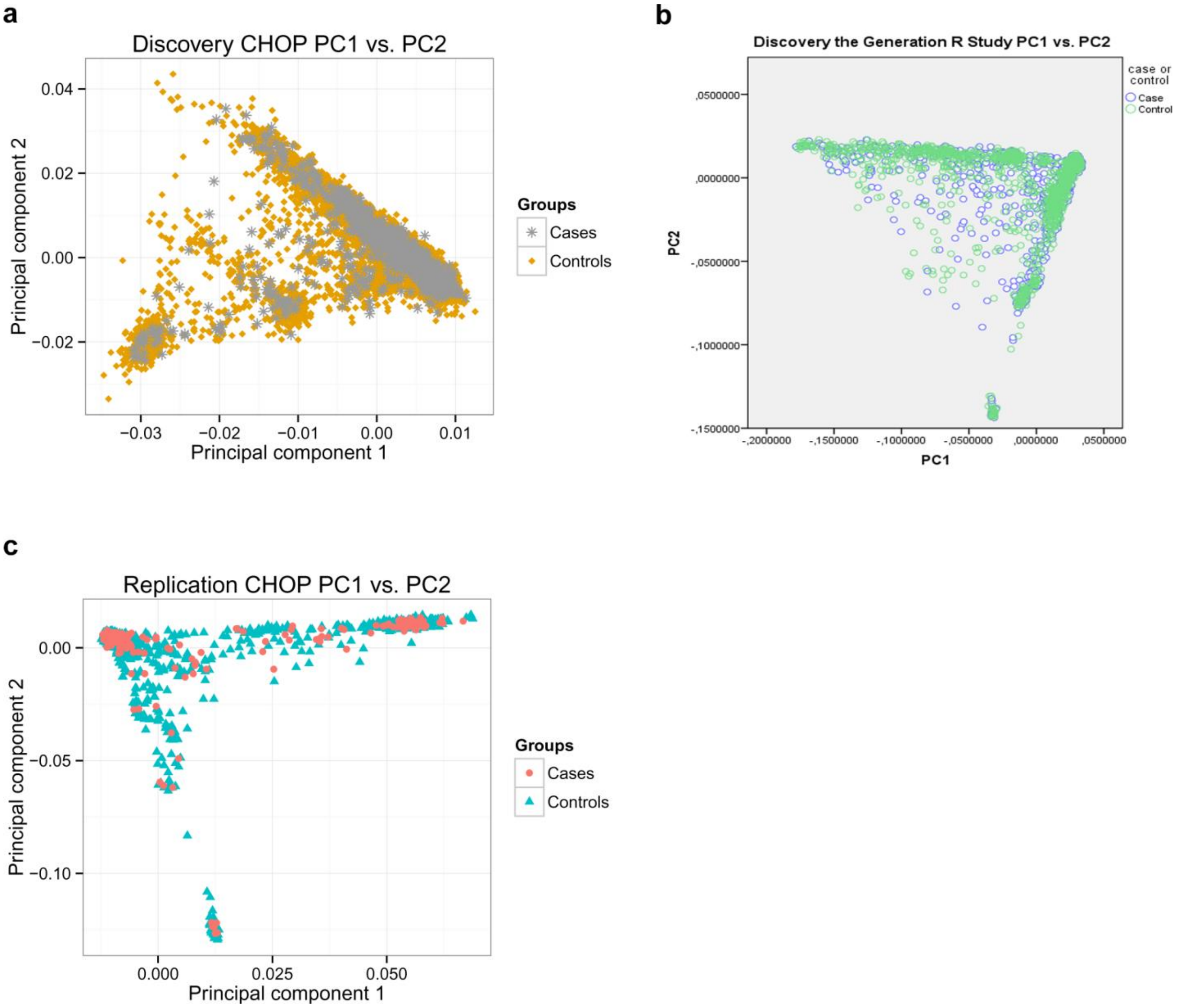

Supplementary Figure 3: Principal component analysis (PCA) of subjects at CHOP and the Generation R Study.

Principal component 1 (PC1) is shown on the X-axis and principal component 2 (PC2) is shown on the Y-axis. (a) PCA results of CHOP discovery cohort; (b) PCA results of the Generation R Study discovery cohort; (c) PCA results of CHOP replication cohort.

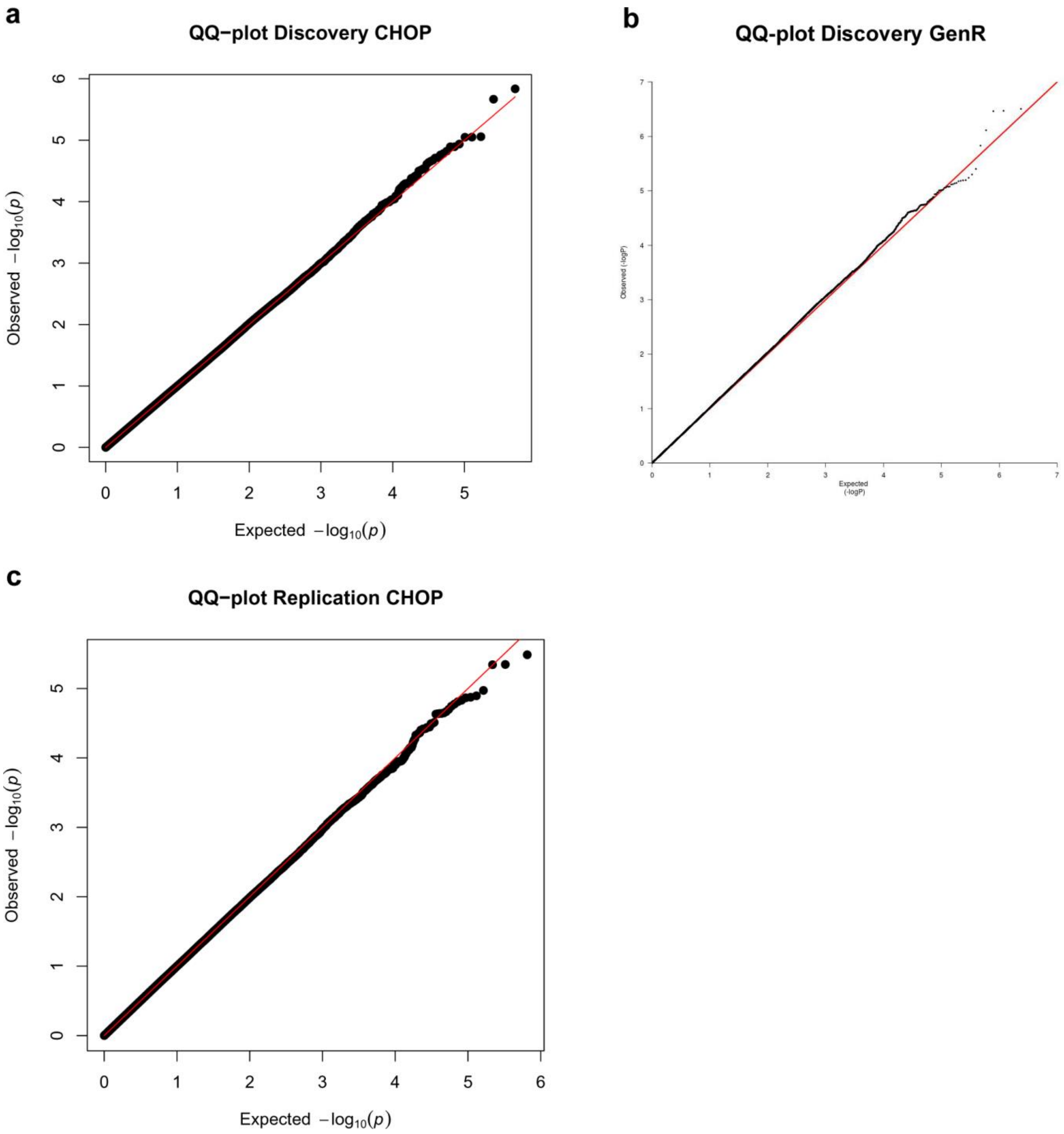

Supplementary Figure 4: Quantile-quantile plot of observed versus expected P-values for each cohort. (a) Discovery CHOP cohort; (b) Discovery cohort of the Generation R Study; (c) Replication CHOP cohort.

Line y = x is shown in red. The genomic inflation factor λ for each cohort is 1.00, 1.01 and 1.01 respectively.

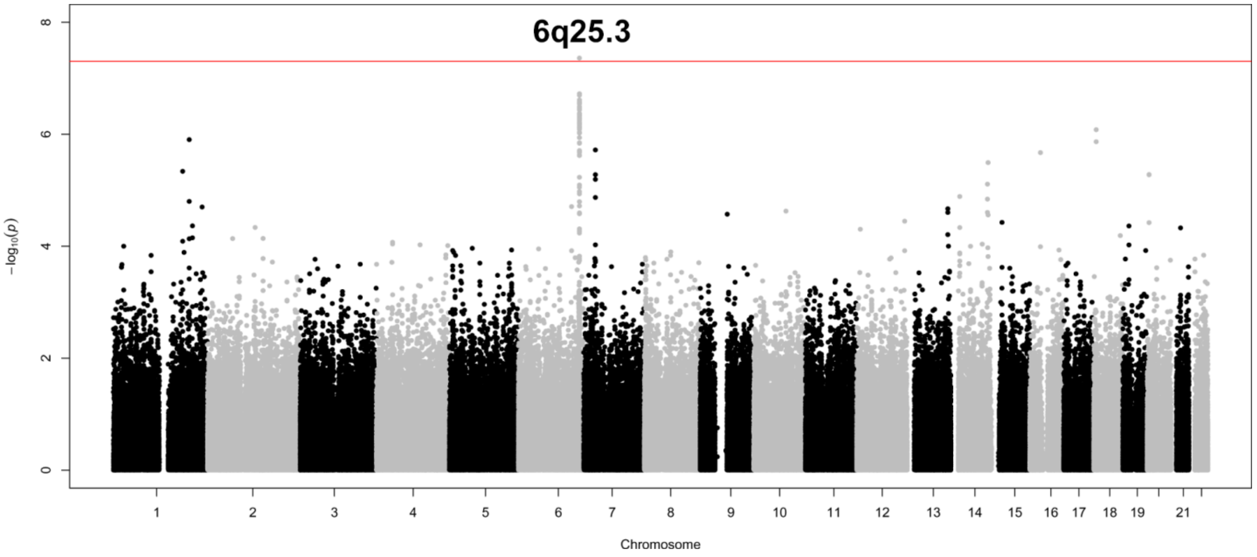

Supplementary Figure 5: Manhattan plot of the association testing statistics showing the AOM associated loci.

The –log10(P-value) of each SNP (Y-axis) is plotted against the SNP genomic position (X-axis). Association statistics were from the meta-analysis of the two cohorts in the discovery phase. The genome-wide significance threshold of $P=5x10^{-8}$ is indicated by the horizontal red line. Both genotyped SNPs and imputed SNPs at 6q25.3 are plotted.

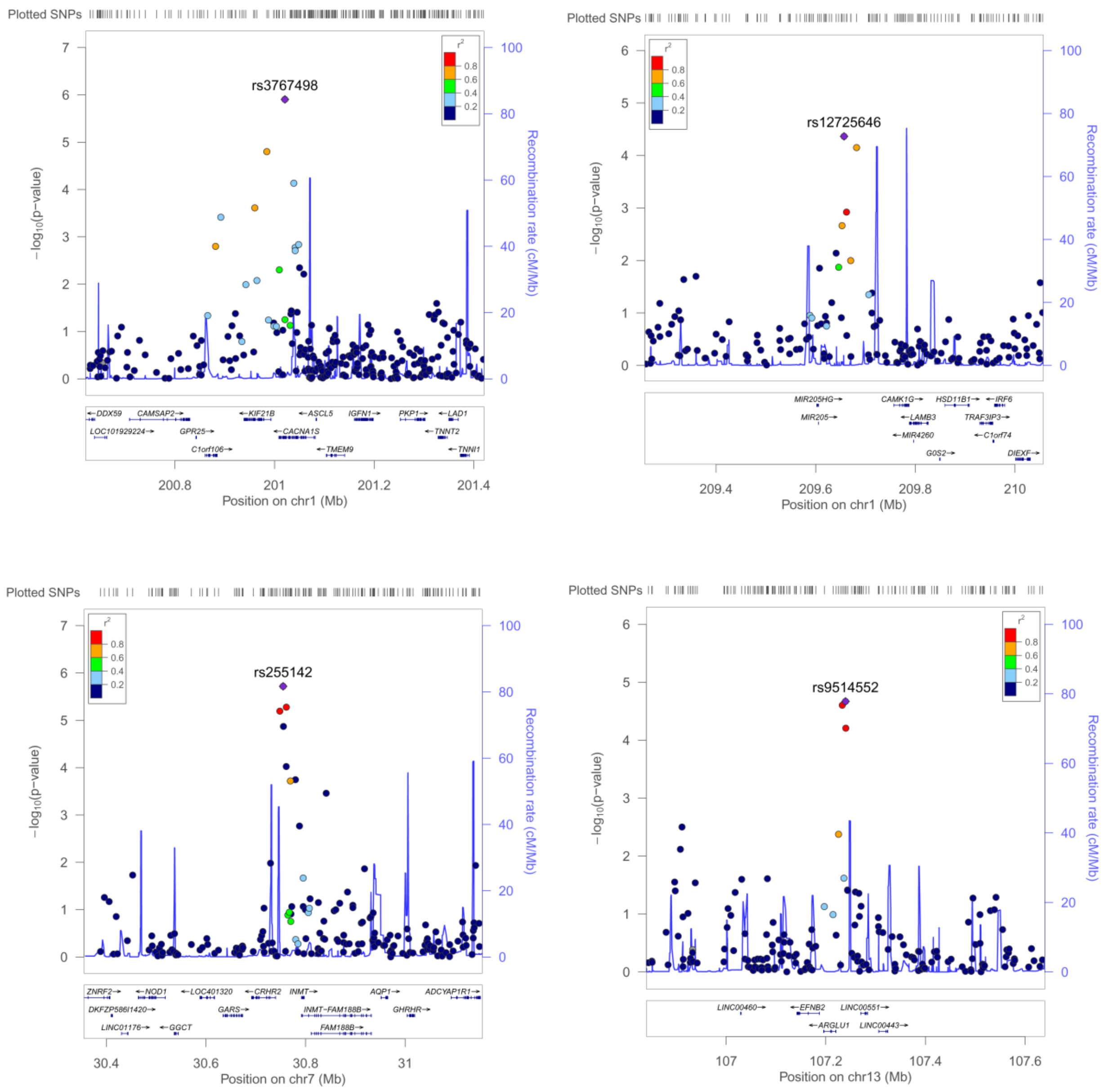
Plotted SNPs
rs3767498
−log10(p−value)
Recombination rate (cM/Mb)
Position on chr1 (Mb)
DDX59
CAMSAP2
KIF21B
ASCL5
IGFN1
PKP1
LAD1
LOC101929224
GPR25
CACNA1S
TNNT2
C1orf106
TMEM9
TNNI1
rs12725646
MIR205HG
CAMK1G
HSD11B1
IRF6
MIR205
LAMB3
TRAF3IP3
MIR4260
C1orf74
G0S2
DIEXF
rs255142
Position on chr7 (Mb)
ZNRF2
NOD1
LOC401320
CRHR2
INMT
AQP1
ADCYAP1R1
DKFZP586I1420
GARS
INMT−FAM188B
GHRHR
LINC01176
GGCT
FAM188B
rs9514552
Position on chr13 (Mb)
LINC00460
EFNB2
LINC00551
ARGLU1
LINC00443

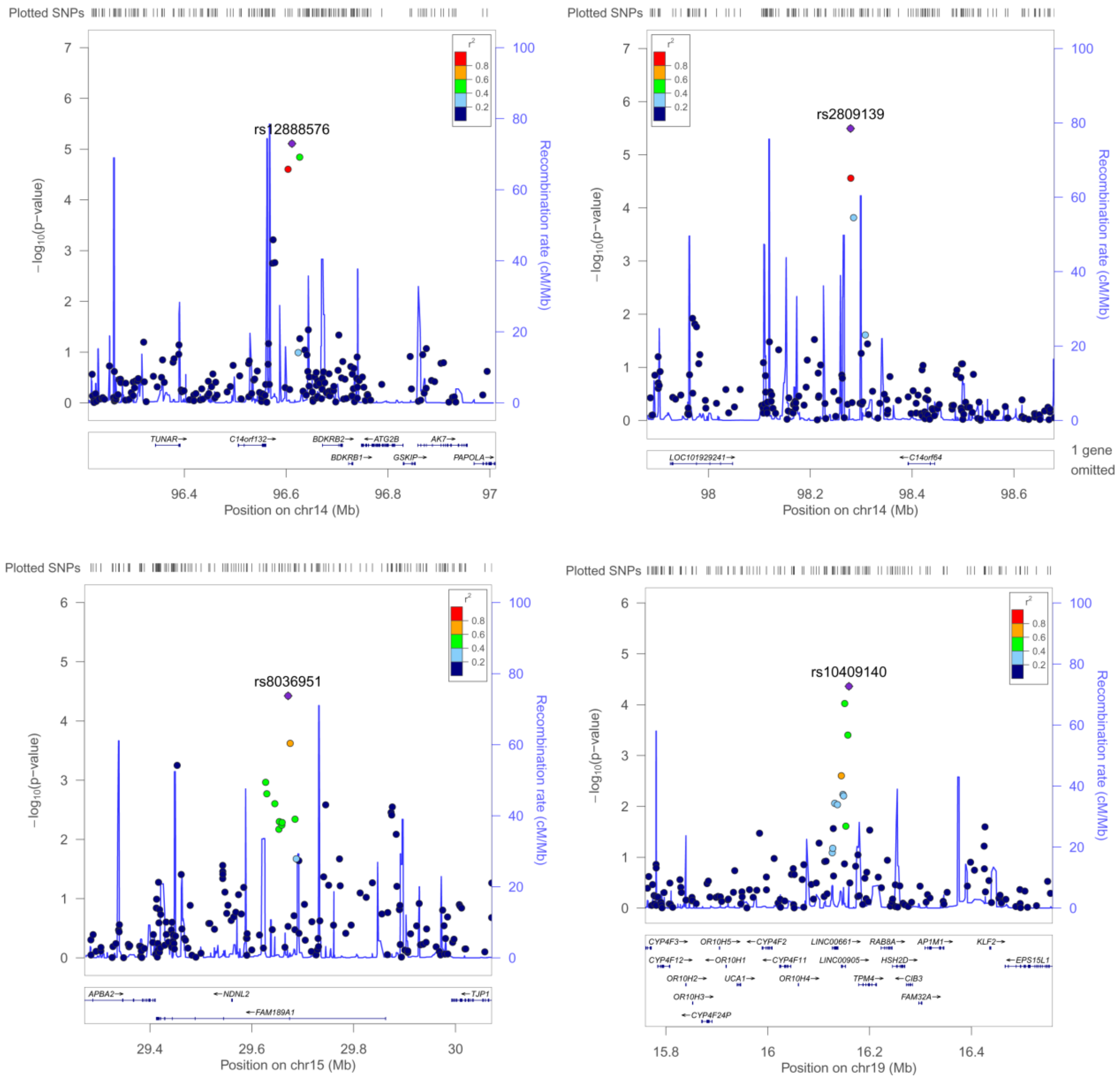
Plotted SNPs
rs12888576
−log10(p−value)
Recombination rate (cM/Mb)
Position on chr14 (Mb)
TUNAR
C14orf132
BDKRB2
ATG2B
AK7
BDKRB1
GSKIP
PAPOLA
Plotted SNPs
rs2809139
−log10(p−value)
Recombination rate (cM/Mb)
LOC101929241
C14orf64
1 gene omitted
Position on chr14 (Mb)
Plotted SNPs
rs8036951
−log10(p−value)
Recombination rate (cM/Mb)
APBA2
NDNL2
TJP1
FAM189A1
Position on chr15 (Mb)
Plotted SNPs
rs10409140
−log10(p−value)
Recombination rate (cM/Mb)
CYP4F3
OR10H5
CYP4F2
LINC00661
RAB8A
AP1M1
KLF2
CYP4F12
OR10H1
CYP4F11
LINC00905
HSH2D
EPS15L1
OR10H2
UCA1
OR10H4
TPM4
CIB3
OR10H3
FAM32A
CYP4F24P
Position on chr19 (Mb)

Supplementary Figure 6: Regional association plots of loci showing suggestive evidence for association ($5x10^{-8}$ < *P*-value < $5x10^{-5}$).

The regional plots were generated using the software LocusZoom[1]. The X-axis indicates the genomic position of the genotyped SNPs, and the Y-axis shows the $–\log_{10}P$-value of these SNPs in our logistic regression analysis. The most strongly associated SNP at each locus is indicated by the purple color, and the colors of the other SNPs represent the strength of linkage disequilibrium with the index SNP. The color coding legend is shown in each plot and the recombination rate is shown by the vertical blue lines.

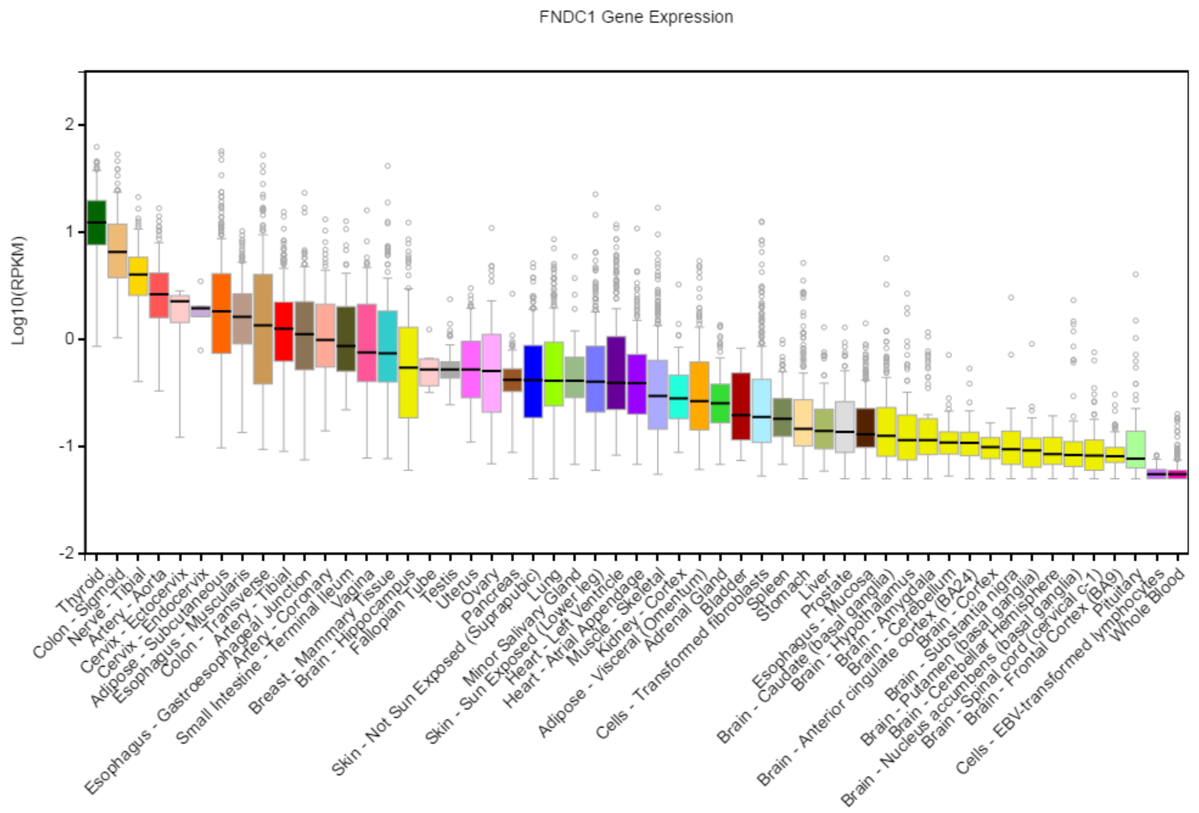

Supplementary Figure 7: Box plot showing the ranking of gene *FNDC1* expression level in different human tissue/cell types.

Tissue/cell types are shown on the X-axis, and the log10 based RPKM (Reads Per Kilobase of transcript per Million mapped reads) values, representing normalized gene expression level, are shown on the Y-axis. The median expression level is shown by the dark black line in each box. The first quartile and the third quartile of the log10(RPKM) are represented by the bottom and the top border of each box, respectively. The lowest and the highest datum within the 1.5 interquartile range (IQR) of the lower quartile and the upper quartile are shown by the end of the lower whisker and that of the upper whisker, respectively. The data points outside of this 1.5 IQR are represented by the grey circles. The plot was generated via GTEx[2] online portal.

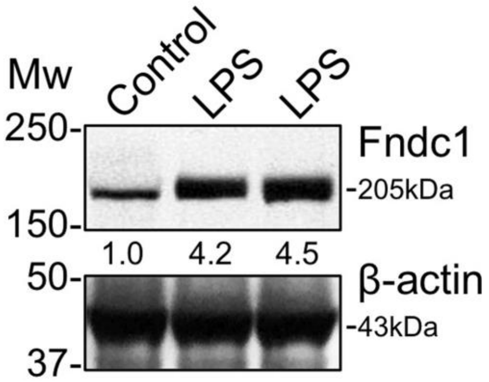

Supplementary Figure 8: The expression of mouse homologue Fndc1 in middle ears and its upregulation upon LPS treatment.

C57/BL6 mice were randomly distributed into two groups. After deep anesthetization, the experimental group received injection into the ears of Lipopolysaccharide (LPS) (2mg per ml) in PBS, 20ul per ear derived from Salmonella typhimurium, and the control group received 0.01M PBS injection. Mice were sacrificed at 3rd day after injection and protein extracts from mice middle ear tissue were separated on a 4–12% NuPAGE Bis-Tris gel and transferred onto nitrocellulose membrane. The upper half of the membrane was probed with rabbit anti-FNDC1 polyclonal antibody; and the lower half of the membrane was probed with rabbit anti-β-Actin polyclonal antibody. Band intensities were measured using Image J software and normalized to β-Actin loading control. A three-fold increase in Fndc1 expression was observed in the experimental group with LPS treatment compared to the control group.

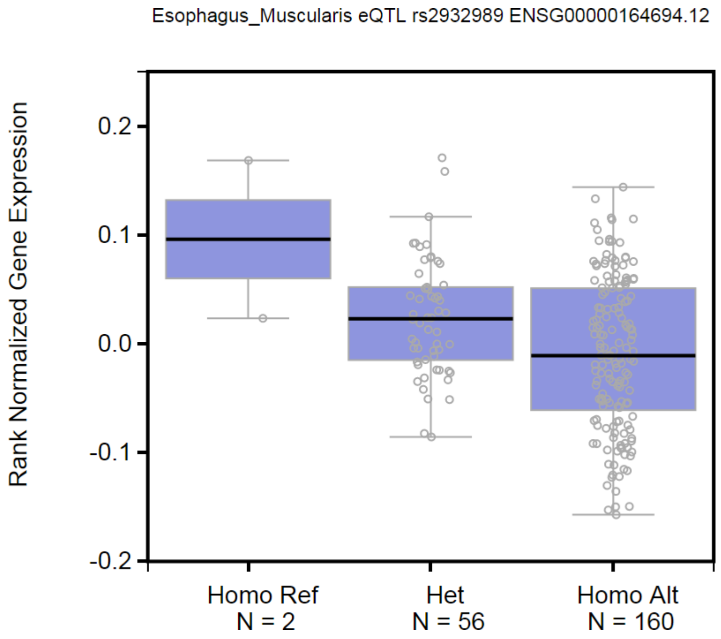

P=9.3x10^{-5}

Supplementary Figure 9: The association between SNP rs2932989 genotype and gene *FNDC1* expression level in tissue esophagus muscularis.

The relationship between SNP rs2932989 genotype and gene *FNDC1* expression level was assessed in GTEx Portal[2]. The different genotype groups of rs2932989 are indicated on the X-axis. In this figure, the reference allele is T and the alternative allele is G. The Rank Normalized gene expression level of FNDC1 is indicated by the Y-axis. The median expression level is shown by the black line in the box plot. The first quartile and the third quartile of the rank normalized gene expression are represented by the bottom and the top border of each box, respectively. The lowest and the highest datum within the 1.5 interquartile range (IQR) of the lower quartile and the upper quartile are shown by the end of the lower whisker and that of the upper whisker, respectively. The data point of each individual is represented by the small grey circle.

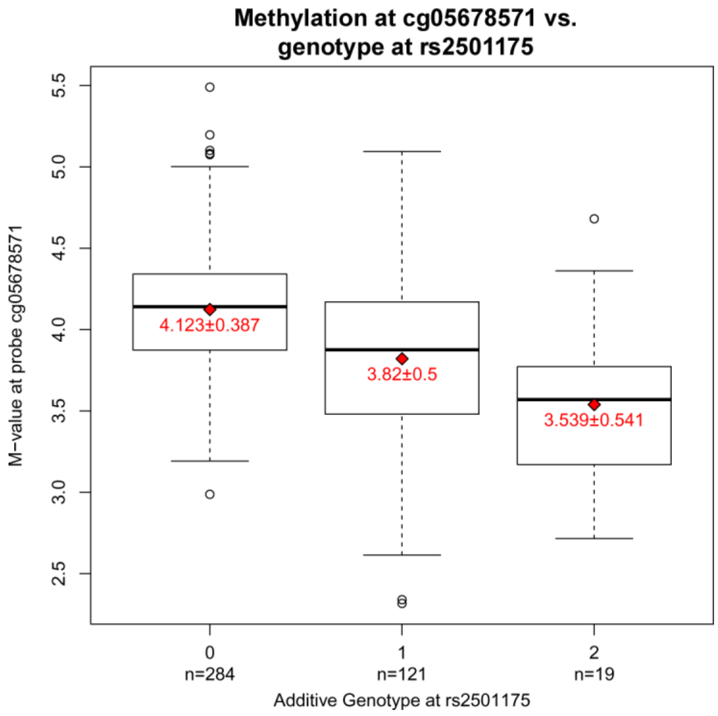

Supplementary Figure 10: The correlation between SNP rs2501175 and methylation probe cg05678571 in gene *FNDC1*.

M-values for methylation probe cg05678571 are plotted against the additive genotype at SNP rs2501175. Dark horizontal lines in the boxplots represent the median of the group, the boxes represent the 25%-75% quantiles, and the whiskers of the boxplot extend beyond those quartiles to 1.5 times the interquartile range. Data outside those ranges are represented by points (open circles). Red diamonds indicate the means of each group, and the red text is the mean ± standard deviation of each group. The number of individuals with each additive genotype of minor allele C is indicated below the x-axis.

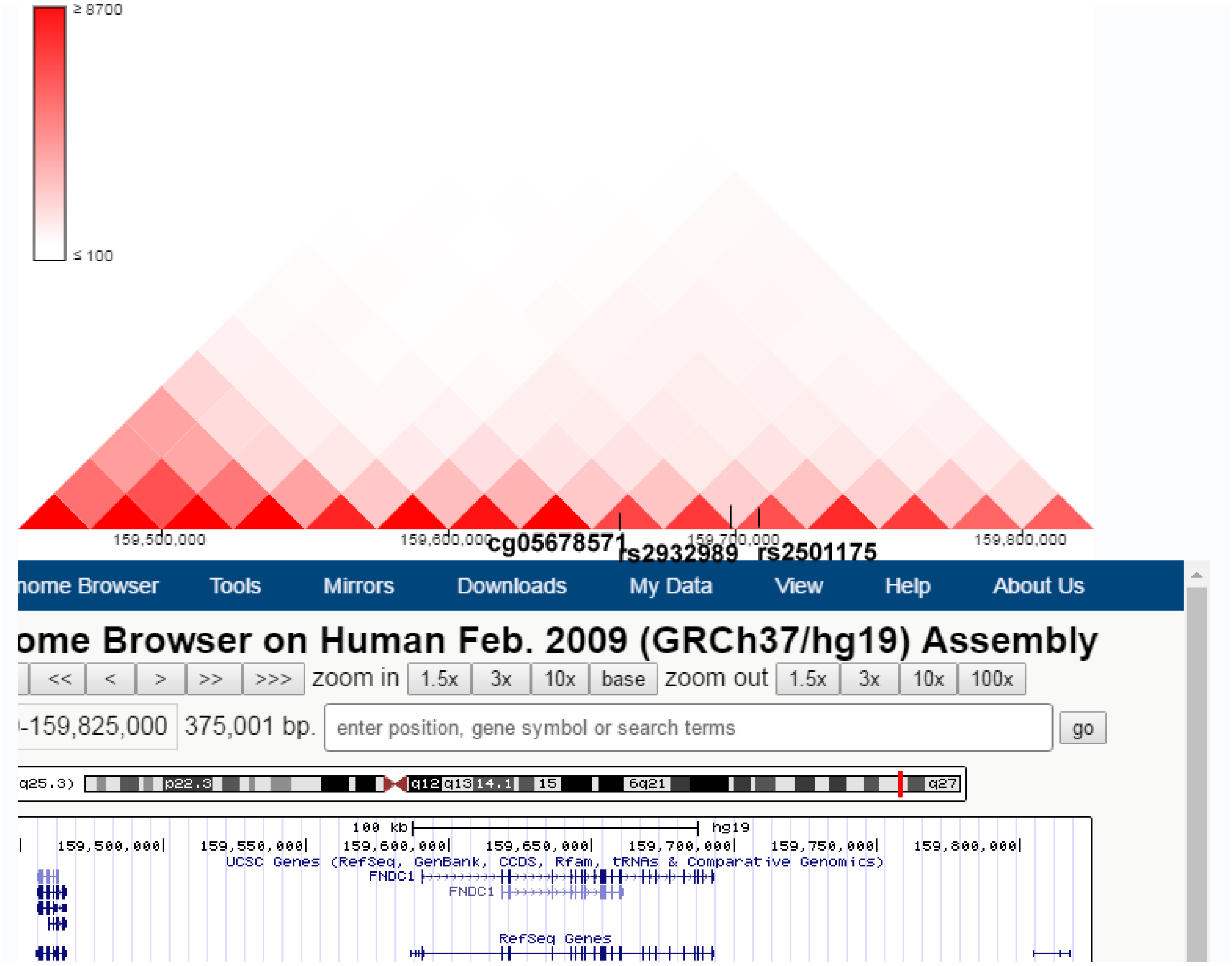

Supplementary Figure 11: The topologically associating domains (TAD) at gene *FNDC1* and surrounding region (chr6:159450000-159825000).

Heatmap shows pairwise interaction frequencies. The genomic location of SNPs rs2932989, rs2501175 and methylation probe cg05678571 is indicated by the black vertical lines.

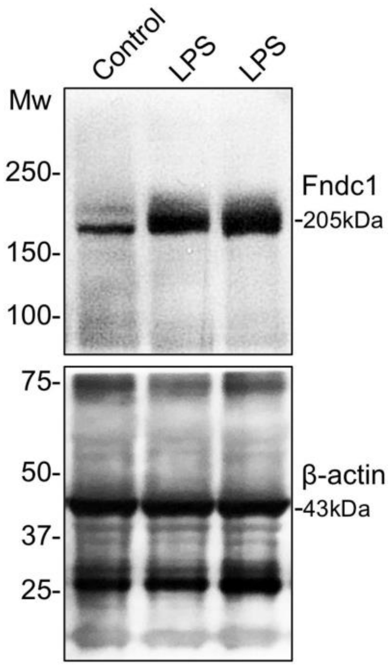

Supplementary Figure 12: Uncropped image of Western blotting in Supplementary Figure 8.

Supplementary Tables

Supplementary Table 1: Phenotype exclusion criteria in case selection of the genome-wide association study. OM cases were excluded from the study if they have any of the following diagnosis.

**Exclusion criteria**
Obstruction of Eustachian tube
Patulous Eustachian tube
Cystic fibrosis*
Primary ciliary dyskinesia♦
Trisomy 21
Cleft palate
Anomalies of skull and face

*or positive CF gene carrier status;
♦or situs inversus;

Supplementary Table 2: The characteristics of study subjects in each cohort after quality control filtering.

**Cohort**		**Control**	**AOM** <3 years
**CHOP Discovery**	N	7,936	825
	♂	4,168 (53%)	504 (61%)
	Age-1st episode+	*	1.3
**Generation R**	N	1,067	1,219
	♂	538 (50%)	656 (54%)
	Age-1st episode+	*	1.2
**CHOP Replication**	N	1,719	293#
	♂	905 (53%)	176 (60%)
	Age-1st episode+	*	NA

*not applicable; +age in years; #AOM; NA – not available

Supplementary Table 3: Association statistics for all SNPs at the locus of 6q25.3 with suggestive evidence of association with AOM in the discovery cohort ($P<5x10^{-05}$).

SNP	Pos (hg19)	A1/A2	OR (95% CI) (CHOP)	P (CHOP)	OR (95% CI) (GenR)	P (GenR)	Direction	P (Meta)	Format*
**rs2932989**	159699284	T/G	1.41 (1.23, 1,62)	$1.46e^{-06}$	1.25 (1.05, 1.48)	$1.02e^{-02}$	++	$4.36e^{-08}$	genotyped
**rs1394232**	159718990	C/G	1.36 (1.19, 1.56)	$3.71e^{-06}$	1.22 (1.03, 1.44)	$1.81e^{-02}$	++	$2.04e^{-07}$	imputed
**rs2221389**	159719183	T/G	1.36 (1.19, 1.56)	$3.71e^{-06}$	1.22 (1.03, 1.44)	$1.81e^{-02}$	++	$2.04e^{-07}$	imputed
**rs1553482**	159722147	T/G	1.33 (1.17, 1.51)	$1.85e^{-06}$	1.17 (1.00, 1.36)	$4.46e^{-02}$	++	$2.46e^{-07}$	imputed
**rs449789**	159699125	C/G	1.35 (1.18, 1.56)	$6.15e^{-06}$	1.24 (1.04, 1.46)	$1.32e^{-02}$	++	$2.55e^{-07}$	imputed
**rs2340806**	159722806	A/G	1.33 (1.17, 1.51)	$2.07e^{-06}$	1.17 (1.00, 1.37)	$4.35e^{-02}$	++	$2.67e^{-07}$	imputed
**rs10806706**	159721945	A/C	1.33 (1.17, 1.51)	$2.02e^{-06}$	1.17 (1.00, 1.36)	$4.47e^{-02}$	++	$2.68e^{-07}$	imputed
**rs4708817**	159720640	A/G	1.33 (1.17, 1.51)	$2.02e^{-06}$	1.17 (1.00, 1.36)	$4.48e^{-02}$	++	$2.69e^{-07}$	imputed
**rs9355713**	159722548	G/A	1.33 (1.17, 1.51)	$2.07e^{-06}$	1.17 (1.01, 1.36)	$4.41e^{-02}$	++	$2.71e^{-07}$	imputed
**rs591953**	159716746	G/A	1.33 (1.17, 1.51)	$2.10e^{-06}$	1.17 (1.00, 1.36)	$4.49e^{-02}$	++	$2.80e^{-07}$	imputed
**rs529281**	159708505	T/C	1.33 (1.17, 1.50)	$2.19e^{-06}$	1.17 (1.01, 1.36)	$4.39e^{-02}$	++	$2.85e^{-07}$	imputed
**rs579533**	159715203	C/T	1.33 (1.17, 1.50)	$2.19e^{-06}$	1.17 (1.01, 1.36)	$4.39e^{-02}$	++	$2.85e^{-07}$	imputed
**rs1875814**	159720266	G/A	1.33 (1.17, 1.51)	$2.15e^{-06}$	1.17 (1.00, 1.36)	$4.49e^{-02}$	++	$2.86e^{-07}$	imputed
**rs1875816**	159720314	A/G	1.33 (1.17, 1.51)	$2.14e^{-06}$	1.17 (1.00, 1.36)	$4.52e^{-02}$	++	$2.87e^{-07}$	imputed
**rs578714**	159715118	G/T	1.33 (1.17, 1.50)	$2.18e^{-06}$	1.17 (1.00, 1.36)	$4.44e^{-02}$	++	$2.87e^{-07}$	imputed
**rs553197**	159714625	G/T	1.33 (1.17, 1.50)	$2.19e^{-06}$	1.17 (1.00, 1.36)	$4.45e^{-02}$	++	$2.89e^{-07}$	imputed
**rs534519**	159711929	A/C	1.33 (1.17, 1.50)	$2.19e^{-06}$	1.17 (1.00, 1.36)	$4.45e^{-02}$	++	$2.89e^{-07}$	imputed
**rs2501175**	159714412	C/T	1.33 (1.17, 1.50)	$2.19e^{-06}$	1.17 (1.00, 1.36)	$4.45e^{-02}$	++	$2.89e^{-07}$	imputed
**rs606833**	159717776	T/A	1.33 (1.17, 1.50)	$2.19e^{-06}$	1.17 (1.00, 1.36)	$4.48e^{-02}$	++	$2.91e^{-07}$	imputed
**rs687314**	159716262	G/C	1.33 (1.17, 1.50)	$2.19e^{-06}$	1.17 (1.00, 1.36)	$4.50e^{-02}$	++	$2.92e^{-07}$	imputed
**rs1687980**	159710460	A/T	1.33 (1.17, 1.50)	$2.19e^{-06}$	1.17 (1.00, 1.36)	$4.99e^{-02}$	++	$3.24e^{-07}$	imputed
**rs473536**	159709843	C/G	1.33 (1.17, 1.50)	$2.19e^{-06}$	1.17 (1.00, 1.36)	$5.25e^{-02}$	++	$3.42e^{-07}$	imputed
**rs531091**	159708697	G/C	1.33 (1.17, 1.50)	$2.19e^{-06}$	1.16 (1.00, 1.36)	$5.64e^{-02}$	++	$3.69e^{-07}$	imputed
**rs294909**	159668662	G/T	1.34 (1.16, 1.54)	$2.16e^{-05}$	1.28 (1.10, 1.49)	$5.00e^{-03}$	++	$4.20e^{-07}$	imputed
**rs824140**	159684911	T/C	1.33 (1.15, 1.54)	$2.96e^{-05}$	1.29 (1.09, 1.54)	$3.37e^{-03}$	++	$4.35e^{-07}$	imputed
**rs633880**	159684584	T/C	1.33 (1.15, 1.54	$2.96e^{-05}$	1.29 (1.09, 1.54)	$3.41e^{-03}$	++	$4.39e^{-07}$	imputed
**rs294902**	159671636	T/C	1.34 (1.16, 1.54)	$2.25e^{-05}$	1.28 (1.08, 1.51)	$5.05e^{-03}$	++	$4.42e^{-07}$	imputed

**rs294896**	159673246	A/G	1.34<br>(1.16, 1.54)	2.25e^{-05}	1.28<br>(1.08, 1.51)	5.08e^{-03}	++	4.44e^{-07}	imputed
**rs534329**	159711857	T/C	1.34<br>(1.16, 1.55)	1.27e^{-05}	1.24<br>(1.05, 1.47)	1.15e^{-03}	++	4.73e^{-07}	imputed
**rs2102244**	159724297	G/A	1.34<br>(1.16, 1.55)	1.15e^{-05}	1.24<br>(1.05, 1.47)	1.33e^{-02}	++	4.83e^{-07}	imputed
**rs2340804**	159713536	C/G	1.34<br>(1.16, 1.55)	1.21e^{-05}	1.24<br>(1.05, 1.46)	1.34e^{-02}	++	5.11e^{-07}	imputed
**rs594613**	159717362	G/C	1.34<br>(1.16, 1.55)	1.27e^{-05}	1.24<br>(1.05, 1.47)	1.33e^{-02}	++	5.35e^{-07}	imputed
**rs372415**	159698312	T/C	1.34<br>(1.16, 1.55)	1.65e^{-05}	1.25<br>(1.06, 1.49)	9.74 e^{-03}	++	5.38e^{-07}	imputed
**rs7760427**	159719239	G/A	1.34<br>(1.16, 1.54)	1.30e^{-05}	1.24<br>(1.05, 1.47)	1.32e^{-02}	++	5.45e^{-07}	imputed
**rs7766678**	159705443	T/C	1.34<br>(1.16, 1.55)	1.29e^{-05}	1.24<br>(1.04, 1.47)	1.43e^{-02}	++	5.77e^{-07}	imputed
**rs625284**	159703373	G/T	1.31<br>(1.16, 1.49)	5.32e^{-06}	1.18<br>(1.01, 1.37)	4.03e^{-02}	++	6.15e^{-07}	imputed
**rs10455775**	159723721	C/T	1.32<br>(1.16, 1.49)	5.03e^{-06}	1.17<br>(1.00, 1.37)	4.27e^{-02}	++	6.17e^{-07}	imputed
**rs568372**	159702765	G/T	1.31<br>(1.16, 1.49)	5.32e^{-06}	1.18<br>(1.01, 1.37)	4.04e^{-02}	++	6.17e^{-07}	imputed
**rs625687**	159703294	G/A	1.31<br>(1.16, 1.49)	5.32e^{-06}	1.18<br>(1.01, 1.37)	4.05e^{-02}	++	6.18e^{-07}	imputed
**rs543547**	159702351	A/G	1.31<br>(1.16, 1.49)	5.32e^{-06}	1.17<br>(1.01, 1.37)	4.05e^{-02}	++	6.19e^{-07}	imputed
**rs575681**	159703534	G/A	1.31<br>(1.16, 1.49)	5.32e^{-06}	1.17<br>(1.01, 1.37)	4.07e^{-02}	++	6.21e^{-07}	imputed
**rs12529478**	159704722	T/C	1.31<br>(1.16, 1.49)	5.32e^{-06}	1.17<br>(1.01, 1.37)	4.30e^{-02}	++	6.56e^{-07}	imputed
**rs7765794**	159704925	T/C	1.32<br>(1.16, 1.49)	5.32e^{-06}	1.17<br>(1.01, 1.37)	4.32e^{-02}	++	6.58e^{-07}	imputed
**rs1687979**	159710299	G/A	1.31<br>(1.16, 1.49)	5.29e^{-06}	1.17<br>(1.01, 1.36)	4.39e^{-02}	++	6.66e^{-07}	imputed
**rs592212**	159711083	G/C	1.31<br>(1.16, 1.49)	5.29e^{-06}	1.17<br>(1.01, 1.36)	4.42e^{-02}	++	6.69e^{-07}	imputed
**rs2451988**	159714390	G/A	1.31<br>(1.16, 1.49)	5.29e^{-06}	1.17<br>(1.00, 1.36)	4.45e^{-02}	++	6.74e^{-07}	imputed
**rs498223**	159707419	A/G	1.34<br>(1.16, 1.54)	1.61e^{-05}	1.24<br>(1.05, 1.46)	1.33e^{-02}	++	6.78e^{-07}	imputed
**rs9346784**	159703757	G/T	1.31<br>(1.16, 1.49)	6.26e^{-06}	1.17<br>(1.01, 1.37)	4.12e^{-02}	++	7.36e^{-07}	imputed
**rs1394233**	159719575	T/C	1.33<br>(1.16, 1.54)	1.77e^{-05}	1.24<br>(1.05, 1.47)	1.33e^{-02}	++	7.46e^{-07}	imputed
**rs578217**	159716957	C/T	1.33<br>(1.16, 1.54)	1.77e^{-05}	1.24<br>(1.05, 1.47)	1.33e^{-02}	++	7.49e^{-07}	imputed
**rs369018**	159694722	A/G	1.33<br>(1.15, 1.53)	2.50e^{-05}	1.26<br>(1.06, 1.50)	8.66e^{-03}	++	7.51e^{-07}	imputed
**rs529273**	159708499	T/C	1.33<br>(1.16, 1.54)	1.87e^{-05}	1.24<br>(1.04, 1.46)	1.36e^{-02}	++	8.05e^{-07}	imputed
**rs509833**	159711515	A/G	1.33<br>(1.15, 1.53)	2.19e^{-05}	1.24<br>(1.05, 1.46)	1.30e^{-02}	++	9.09e^{-07}	imputed
**rs650456**	159713117	T/C	1.33<br>(1.17, 1.50)	2.19e^{-06}	1.13<br>(0.96, 1.33)	0,132	++	9.50e^{-07}	imputed
**rs419009**	159692596	T/C	1.32<br>(1.14, 1.53)	4.74e^{-05}	1.28<br>(1.08, 1.53)	5.14e^{-03}	++	9.79e^{-07}	imputed
**rs1394234**	159732990	A/G	1.31<br>(1.14, 1.51)	2.02e^{-05}	1.21<br>(1.02, 1.43)	2.43e^{-02}	++	1.43e^{-06}	imputed
**rs434578**	159693220	C/T	1.32<br>(1.14, 1.52)	4.25e^{-05}	1.25<br>(1.06, 1.49)	9.83e^{-03}	++	1.44e^{-06}	imputed
**rs389546**	159691657	T/C	1.32<br>(1.14, 1.53)	6.89e^{-05}	1.28<br>(1.08, 1.53)	5.11e^{-03}	++	1.45e^{-06}	imputed

**rs7767146**	159728226	T/C	1.31 (1.14, 1.50)	$2.35e^{-05}$	1.20 (1.02, 1.41)	$2.79e^{-02}$	++	$1.88e^{-06}$	imputed
**rs4709297**	159731869	G/A	1.31 (1.14, 1.50)	$2.35e^{-05}$	1.20 (1.02, 1.41)	$2.83e^{-02}$	++	$2.05e^{-06}$	imputed
**rs7774311**	159726752	G/A	1.31 (1.14, 1.50)	$2.57e^{-05}$	1.20 (1.02, 1.41)	$2.79e^{-02}$	++	$2.05e^{-06}$	imputed
**rs1394235**	159728693	G/A	1.31 (1.14, 1.50)	$2.53e^{-05}$	1.20 (1.02, 1.41)	$2.84e^{-02}$	++	$2.05e^{-06}$	imputed
**rs1504255**	159731221	A/G	1.31 (1.14, 1.50)	$2.53e^{-05}$	1.20 (1.02, 1.40)	$2.91e^{-02}$	++	$2.10e^{-06}$	imputed
**rs905684**	159733694	T/G	1.34 (1.17, 1.53)	$2.45e^{-05}$	1.19 (1.01, 1.39)	$3.44e^{-02}$	++	$2.36e^{-06}$	genotyped
**rs4077944**	159734491	T/C	1.30 (1.13, 1.50)	$2.99e^{-05}$	1.20 (1.02, 1.41)	$2.86e^{-02}$	++	$2.44e^{-06}$	imputed
**rs76252099**	159659971	A/G	1.35 (1.15, 1.59)	$1.22e^{-04}$	1.32 (1.06, 1.65)	$1.48e^{-02}$	++	$5.88e^{-06}$	imputed
**rs76096006**	159663831	G/C	1.33 (1.14, 1.59)	$1.82e^{-04}$	1.33 (1.06, 1.66)	$1.33e^{-02}$	++	$8.19e^{-06}$	imputed
**rs79695409**	159663703	T/C	1.33 (1.14, 1.59)	$1.83e^{-04}$	1.33 (1.06, 1.66)	$1.33e^{-02}$	++	$8.25e^{-06}$	imputed
**rs381568**	159639482	T/C	1.26 (1.13, 1.40)	$3.87e^{-05}$	1.12 (0.98, 1.27)	$8.82e^{-02}$	++	$8.84e^{-06}$	genotyped
**rs77131924**	159655516	C/T	1.33 (1.14, 1.59)	$2.15e^{-04}$	1.32 (1.06, 1.65)	$1.45e^{-02}$	++	$1.05e^{-05}$	imputed
**rs59701391**	159656107	A/G	1.33 (1.14, 1.56)	$2.15e^{-04}$	1.32 (1.06, 1.65)	$1.46e^{-02}$	++	$1.05e^{-05}$	imputed
**rs73799724**	159658029	G/C	1.33 (1.14, 1.56)	$2.48e^{-04}$	1.32 (1.06, 1.65)	$1.35e^{-02}$	++	$1.15e^{-05}$	imputed
**rs7744848**	159770787	G/A	1.36 (1.18, 1.57)	$1.74e^{-05}$	1.10 (0.93, 1.30)	0.282	++	$1.60e^{-05}$	genotyped
**rs76029050**	159723266	T/C	1.37 (1.14, 1.64)	$4.33e^{-04}$	1.35 (1.07, 1.71)	$1.19e^{-02}$	++	$1.89e^{-05}$	imputed
**rs75391241**	159671268	G/A	1.35 (1.14, 1.61)	$5.67e^{-04}$	1.34 (1.06, 1.69)	$1.26e^{-02}$	++	$2.57e^{-05}$	imputed
**rs7746188**	159754888	A/G	1.30 (1.13, 1.50)	$3.54e^{-05}$	1.11 (0.93, 1.31)	0.248	++	$2.57e^{-05}$	imputed
**rs1875819**	159747743	T /C	1.29 (1.14, 1.47)	$8.07e^{-05}$	1.10 (0.94, 1.29)	0.227	++	$4.90e^{-05}$	genotyped

SNP – single nucleotide polymorphism; Pos – position; A1 – effect allele; A2 – non-effect allele; OR – odds ratio; CI – confidence interval; P - association P-value per cohort and in meta-analysis; Direction – direction of effect in both discovery analyses; Format*: SNPs imputed in regional imputation analysis are indicated as “imputed”.

Supplementary Table 4: Replication cohort association results for SNPs that are in strong LD with rs2932989 and are on chip HumanOmniExpress-12v1.

SNP	Chr	Pos (hg19)	A1/A2	OR	95% CI	P	r^2
**rs419009**	6	159692596	T/C	1.39	1.07, 1.80	0.012	1
**rs578217**	6	159716957	C/T	1.38	1.06, 1.79	0.016	1

SNP – single nucleotide polymorphism; Chr – chromosome; Pos – position; A1 – minor allele; A2 – major allele; OR – odds ratio; CI – confidence interval; P – association P-value in CHOP replication cohort; r^2 – LD measurement between each SNP and rs2932989 in 1000Genome CEU dataset.

Supplementary Table 5: Summary statistics of index SNP at each loci with suggestive evidence for association (5×10^{-8} < P-value < 5×10^{-5}).

SNP	Chr	Pos (hg19)	Nearby Genes	Distance (bp)	A1/A2	P_{meta}	Direction
**rs3767498**	1	201020727	*KIF21B, CACNA1S, ASCL5*	0	A/G	$1.25x10^{-06}$	++
**rs12725646**	1	209656906	intergenic region near *MIR205HG*	51014	C/T	$4.33x10^{-05}$	++
**rs255142**	7	30754949	*CRHR2, INMT*	32809	A/G	$1.91x10^{-06}$	++
**rs9514552**	13	107239714	*ARGLU1*	19200	G/A	$2.14x10^{-05}$	--
**rs12888576**	14	96611391	*BDKRB2*	59744	G/A	$7.79x10^{-06}$	++
**rs2809139**	14	98279189	intergenic region near *C14orf177*	898761	C/T	$3.21x10^{-06}$	++
**rs8036951**	15	29671295	*FAM189A1*	0	G/A	$3.76x10^{-05}$	++
**rs10409140**	19	16159493	*TPM4*	27832	A/G	$4.35x10^{-05}$	--

SNP – single nucleotide polymorphism; Chr – chromosome; Pos – position (hg19); Distance – distance to closest gene in base-pairs; A1 – effect allele; A2 – non-effect allele; P_{meta} - association P-value in meta-analysis; Direction – direction of effect in both discovery analyses.

Supplementary Table 6. Association statistics of candidate genes previously proposed to be involved in pathogenesis of otitis media.

Each SNP in our GWAS is mapped to its closest gene. For genes the best SNP of which is located outside of its transcript or upstream/downstream 50kb region, the top SNP within this defined gene boundary is also shown. Nominally significant *P*-values are highlighted.

Gene	Best SNP	Chr	Pos (hg19)	Distance	A1/A2	P_meta	Direction	Study type	Reference
TP73*	rs3765731	1	3611892	0	A/G	**0.0274**	--	Candidate gene	3
CCDC27	rs1181876	1	3681681	0	T/C	0.0724	--	GWAS	4
LIN28	rs11581746	1	26744087	0	T/C	**0.0429**	--	GWAS	4
CLCA1*	rs2734700	1	86950320	0	A/G	**0.00383**	--	Candidate gene	5
OVGP1	rs1777833	1	111956473	464	G/A	0.176	-+	Candidate gene	6
MUC1	rs4072037	1	155162067	0	G/A	0.904	-+	Candidate gene	7
IL10	rs3024505	1	206939904	1044	T/C	0.277	--	Candidate gene	8
PARP1	rs1136410	1	226555302	0	C/T	**0.0162**	-+	Candidate gene	9
GALNT14	rs4245766	2	31242194	0	C/T	**0.0170**	++	GWAS	4
CAPN14	rs17010826	2	31396729	0	A/G	**0.0343**	+-	GWAS	4
FBXO11(*)	rs4952896	2	48116126	0	G/A	0.244	++	Candidate gene	10, 11
FBXO11(*)	rs999036	2	48217552	84738	G/A	**0.0466**	++	Candidate gene	10, 11
IL1A	rs17042407	2	113558914	15943	C/T	0.492	--	Candidate gene	12
IL1B	rs2723167	2	113621210	26854	T/C	**0.0283**	--	Candidate gene	13
IL1RN	rs315952	2	113890304	0	C/T	**0.0475**	--	Transcriptome analysis	14
GALNT13	rs13414624	2	154904730	0	A/C	**0.0141**	--	GWAS	4
CDCA7	rs6751320	2	174264424	30706	T/C	**0.0464**	++	GWAS	15
CDCA7	rs6714145	2	174454044	220326	C/T	**0.00257**	--	GWAS	15
SP3	rs4972618	2	174769344	3914	T/C	**0.0299**	--	GWAS	15
IRAK2	rs6442159	3	10243304	0	A/G	0.147	+-	Candidate gene	16, 17
HRH1	rs443137	3	11253721	0	T/C	**0.0373**	++	Genome scan	17
MYD88*	rs7744	3	38184021	0	G/A	0.147	+-	Candidate gene	18
LTF	rs17078876	3	46500978	0	T/C	0.403	++	Candidate gene	19
IL12A	rs550662	3	159663317	43312	A/C	**0.00396**	++	Candidate gene	20
EVI1*	rs10513653	3	168746566	54721	A/G	**0.00966**	++	Candidate gene	21
EVI1*	rs6798744	3	168791596	9691	C/T	0.09187	--	Candidate gene	21
BCL6	rs2229362	3	187446211	0	A/G	0.227	--	Candidate gene	22
BCL6	rs2889879	3	187657216	193741	C/T	**0.0181**	++	Candidate gene	22
MUC4	rs2259419	3	195505417	0	G/A	**0.0115**	--	Candidate gene	7
IDUA*	rs3755955	4	994414	0	T/C	0.191	--	Candidate gene	23
MUC7	rs6600832	4	71353508	4794	C/T	0.122	--	Candidate gene	6
IL8	rs12506479	4	74592161	14114	C/T	0.259	--	Candidate gene	24
GRID2	rs977955	4	93966480	0	A/G	**0.00168**	--	GWAS	4
IL2*	rs10857092	4	123389219	11569	A/G	0.577	-+	Candidate gene	25
TLR2	rs1816702	4	154609523	0	T/C	0.288	--	Candidate gene	26
TPPP	rs413666	5	673408	0	T/C	0.376	+-	GWAS	15

DNAH5	rs1910092	5	13781480	0	T/C	**0.0241**	++	Candidate gene	27
ISL1*	rs2961811	5	50413703	265255	A/C	**0.00269**	++	exome sequencing	28
ISL1*	rs10939961	5	50738478	47915	A/C	0.319	++	exome sequencing	28
IL13	rs1295686	5	131995843	0	A/G	**0.0266**	--	Candidate gene	29
CD14	rs778584	5	140005212	6105	T/C	0.0661	-+	Candidate gene	30
IL12B	rs2569253	5	158750993	0	C/T	**0.0152**	--	Candidate gene	20
SH3PXD2B*	rs10059733	5	171908794	27267	G/A	0.110	++	Candidate gene	31
SH3PXD2B*	rs7713320	5	172024642	143115	T/C	**0.0117**	++	Candidate gene	31
TNF	rs3093662	6	2846777	0	G/A	0.974	--	Candidate gene	32
SPDEF*	rs13194133	6	34532922	8831	G/T	0.121	--	Candidate gene	5
BMP5	rs9475400	6	55638258	0	T/C	**0.0119**	--	GWAS	4
EYA4*	rs211619	6	133727321	0	A/G	**0.00226**	++	Candidate gene	33
PLG*	rs6935921	6	161108536	14738	C/T	0.236	++	Candidate gene	34
DNAH11*	rs1859107	7	21859436	0	C/A	**0.0166**	--	Candidate gene	35
IL6	rs10261484	7	22583326	183492	G/A	**0.0416**	++	Candidate gene	32
IL6	rs6949149	7	22749157	17661	T/G	0.0877	--	Candidate gene	32
GUSB*	rs12698511	7	65474919	27673	A/C	0.150	--	Candidate gene	36
DEFB1	rs2702839	8	6722082	6017	A/G	**0.0133**	--	Candidate gene	37
CTSB	rs12898	8	11701198	0	C/T	0.306	--	Candidate gene	38
FGFR1(*)	rs6996321	8	38322346	0	A/G	**0.00163**	++	Candidate gene	39, 40
TLR4(*)	rs4986790	9	120475302	0	G/A	0.0912	++	Candidate gene	41, 42
TLR4(*)	rs955259	9	120904985	425219	C/T	**0.0111**	++	Candidate gene	41, 42
ADAMTS13	rs685523	9	136310908	0	A/G	**0.0347**	-+	Case report	43
MBL2	rs2120132	10	54526040	0	C/T	**0.0213**	--	Candidate gene	44
MBL2	rs1962474	10	54914951	383491	C/T	**0.0133**	++	Candidate gene	44
SFTPA1	rs1650198	10	81417214	42012	C/T	0.495	-+	Candidate gene	45
SFTPD	rs1885551	10	81712353	3492	C/T	0.125	+-	Candidate gene	41
SFTPD	rs1932571	10	81761489	52628	T/C	**0.0346**	++	Candidate gene	41
FAS*	rs9325604	10	90775756	214	G/A	**0.00419**	-+	Candidate gene	46
FAS*	rs9325604	10	90775756	214	G/A	**0.00419**	-+	Candidate gene	46
ADAM8	rs1131718	10	135085754	0	C/T	0.595	--	Candidate gene	17
MUC6	rs11246381	11	1019194	0	G/A	**0.0105**	--	Candidate gene	6
MUC2	rs4077759	11	1105976	1559	C/T	0.0789	++	Candidate gene	47
MUC5AC	rs868903	11	1242690	0	T/C	0.357	-+	Candidate gene	48
MUC5AC,MUC5B	rs2075859	11	1250488	1	T/C	0.295	--	Candidate gene	48
MUC5AC,MUC5B	rs2075859	11	1250488	0	T/C	0.295	--	Candidate gene	48
NELL1	rs11026076	11	21436232	0	T/C	**0.00132**	--	GWAS	4
A2ML1	rs16917857	12	9020680	0	A/G	**0.0319**	--	Exome sequencing	49
IFNG	rs1548653	12	68360943	187607	G/A	0.114	--	Candidate gene	50
IFNG	rs2193045	12	68534520	14030	A/G	0.352	+-	Candidate gene	50
OXGR1*	rs10851069	13	97628279	9694	T/C	0.0591	++	Candidate gene	51
TGFB3	rs3917192	14	76431674	0	A/G	0.311	++	GWAS	4
SMAD3	rs266377	15	67229636	128559	G/T	0.0507	--	Candidate gene	52
SMAD3	rs893473	15	67438091	0	T/C	0.102	++	Candidate gene	52
TICRR	rs8041127	15	90135196	0	A/G	0.407	-+	GWAS	15
KIF7	rs1110060	15	90190048	0	A/G	0.771	--	GWAS	15
MMP2	rs243866	16	55511537	1544	A/G	**0.0234**	++	Candidate gene	53

E2F4*	rs11700	16	67232684	0	C/T	0.315	-+	Candidate gene	[54]
E2F4*	rs11700	16	67232684	0	C/T	0.315	-+	Candidate gene	[54]
CCL3*	rs1851503	17	34412202	3401	G/T	0.878	+-	Candidate gene	[55]
RPL38*	rs6501659	17	71771387	428408	T/C	**0.035**	++	Candidate gene	[56]
RPL38*	rs7211140	17	72173910	25885	A/G	0.0916	-+	Candidate gene	[56]
SMAD2	rs1942159	18	45484940	27428	G/A	**0.000919**	--	Candidate gene	[57]
SMAD4	rs948588	18	48586344	0	A/G	**0.0342**	--	Candidate gene	[57]
TICAM1*	rs8120	19	4816160	0	G/T	**0.0367**	++	Candidate gene	[58]
TGFB1	rs8110090	19	41845872	0	G/A	0.41	+-	Candidate gene	[59]
FOXA2*	rs199820	20	22323038	238604	A/C	**0.0022**	--	Candidate gene	[60]
FOXA2*	rs6075924	20	22512529	49113	T/C	0.122	--	Candidate gene	[60]
MMP9	rs13038175	20	44624097	13450	A/G	0.384	++	Candidate gene	[53]
SALL4*	rs6063719	20	50440054	21006	T/G	**0.0391**	+-	Candidate gene	[61]
TBX1*	rs4819843	22	19765182	0	A/G	0.396	++	Candidate gene	[62]
NF2*	rs2530664	22	30038152	0	C/A	**0.0342**	++	Candidate gene	[63]
CBY1*	rs4821797	22	39016182	36476	C/T	0.429	--	Candidate gene	[64]

* the rodent model homologue of which has been implicated in otitis media
Best SNP: The SNP of the lowest P-value in each gene; Chr: chromosome; Pos(hg19): the position on human genome build hg19; Distance: the distance between the SNP and the gene transcript; A1: effect allele; A2: non-effect allele; P_meta: meta-analysis P-value; Direction: the direction of effect for the effect allele in the two cohorts of meta-analysis, "+" represents risk effect, "-" represents protective effect; Study type: the type of the study reported in the reference.

Supplementary Table 7: Summary of mouse gene Fndc1 tissue expression pattern.

Data are from http://www.informatics.jax.org/

Structure	Total number of assays	Number of assays Detected	Level	References
**TSTS21: eye**	1	1	NA	[65]
**TSTS22: conjunctival sac**	1	1	NA	[65]
**TSTS23: conjunctival sac**	4		Strong	[66]
**TSTS23: cornea epithelium**	5	5	Strong	[66]
**TSTS23: oral epithelium**	16	16	Strong	[66]
**TSTS23: sublingual gland primordium**	5	5	Strong	[66]
**TSTS23: submandibular gland primordium**	7	7	Strong	[66]
**TSTS23: dermis**	24	24	Strong	[66]
**TSTS23: lower lip**	7	7	Strong	[66]
**TSTS23: upper lip**	9	9	Strong	[66]
**TSTS23: mesenchyme**	24	24	Strong	[66]
**TSTS24: conjunctival sac**	1	1	NA	[65]
**TSTS26: conjunctival sac**	1	1	NA	[65]
**TSTS27: conjunctival sac**	1	1	NA	[65]

NA: not reported.

Supplementary Table 8: Proteins bound to significant SNPs at locus 6q25.3.

We input all SNPs of suggestive significant association with AOM ($P<5x10^{-5}$) into Haploreg search[67]. Two of them were found to have proteins bound in ENCODE ChIP assay[68].

SNP	Chr	Pos (hg19)	Proteins bound	Cell lines
**rs1394234**	6	159732990	CTCF	AG10803<br>GM06990<br>GM12878<br>NHEK
**rs1553482**	6	159722147	EGR1	K562

SNP – single nucleotide polymorphism; Chr – chromosome; Pos – position; AG10803 – abdominal skin fibroblasts; GM06990 – B-lymphocyte, lymphoblastoid; GM12878 – lymphoblastoid cell line; NHEK – Normal Human Epidermal Keratinocytes; K562 – leukemia.

Supplementary References

1. Pruim RJ*, et al.* LocusZoom: regional visualization of genome-wide association scan results. *Bioinformatics* **26**, 2336-2337 (2010).

2. The GTEx Consortium. The Genotype-Tissue Expression (GTEx) pilot analysis: multitissue gene regulation in humans. *Science* **348**, 648-660 (2015).

3. Yang A*, et al.* p73-deficient mice have neurological, pheromonal and inflammatory defects but lack spontaneous tumours. *Nature* **404**, 99-103 (2000).

4. Rye MS*, et al.* Genome-wide association study to identify the genetic determinants of otitis media susceptibility in childhood. *PLoS One* **7**, e48215 (2012).

5. Nakamura Y, Hamajima Y, Komori M, Yokota M, Suzuki M, Lin J. The role of atoh1 in mucous cell metaplasia. *International journal of otolaryngology* **2012**, 438609 (2012).

6. Takeuchi K, Yagawa M, Ishinaga H, Kishioka C, Harada T, Majima Y. Mucin gene expression in the effusions of otitis media with effusion. *Int J Pediatr Otorhinolaryngol* **67**, 53-58 (2003).

7. Tsuboi Y, Kim Y, Paparella MM, Chen N, Schachern PA, Lin J. Pattern changes of mucin gene expression with pneumococcal otitis media. *Int J Pediatr Otorhinolaryngol* **61**, 23-30 (2001).

8. Alper CM, Winther B, Hendley JO, Doyle WJ. Cytokine polymorphisms predict the frequency of otitis media as a complication of rhinovirus and RSV infections in children. *European archives of oto-rhino-laryngology : official journal of the European Federation of Oto-Rhino-Laryngological Societies* **266**, 199-205 (2009).

9. Esposito S*, et al.* Genetic Polymorphisms of Functional Candidate Genes and Recurrent Acute Otitis Media With or Without Tympanic Membrane Perforation. *Medicine* **94**, e1860 (2015).

10. Segade F*, et al.* Association of the FBXO11 gene with chronic otitis media with effusion and recurrent otitis media: the Minnesota COME/ROM Family Study. *Archives of otolaryngology--head & neck surgery* **132**, 729-733 (2006).

11. Hardisty-Hughes RE*, et al.* A mutation in the F-box gene, Fbxo11, causes otitis media in the Jeff mouse. *Human molecular genetics* **15**, 3273-3279 (2006).

12. Joki-Erkkila VP, Puhakka H, Hurme M. Cytokine gene polymorphism in recurrent acute otitis media. *Archives of otolaryngology--head & neck surgery* **128**, 17-20 (2002).

13. McCormick DP*, et al.* Acute otitis media severity: association with cytokine gene polymorphisms and other risk factors. *Int J Pediatr Otorhinolaryngol* **75**, 708-712 (2011).

14. Hernandez M, Leichtle A, Pak K, Webster NJ, Wasserman SI, Ryan AF. The transcriptome of a complete episode of acute otitis media. *BMC genomics* **16**, 259 (2015).

15. Allen EK*, et al.* A genome-wide association study of chronic otitis media with effusion and recurrent otitis media identifies a novel susceptibility locus on chromosome 2. *J Assoc Res Otolaryngol* **14**, 791-800 (2013).

16. Lee HY, *et al.* Induction of beta defensin 2 by NTHi requires TLR2 mediated MyD88 and IRAK-TRAF6-p38MAPK signaling pathway in human middle ear epithelial cells. *BMC infectious diseases* **8**, 87 (2008).

17. Daly KA, *et al.* Chronic and recurrent otitis media: a genome scan for susceptibility loci. *Am J Hum Genet* **75**, 988-997 (2004).

18. Hernandez M, *et al.* Myeloid differentiation primary response gene 88 is required for the resolution of otitis media. *J Infect Dis* **198**, 1862-1869 (2008).

19. Suzuki T, Somekawa Y, Yamanaka N, Niida Y, Kataura A. Lactoferrin in middle ear effusion. *Auris, nasus, larynx* **12 Suppl 1**, S154-155 (1985).

20. Kariya S, *et al.* TH1/TH2 and regulatory cytokines in adults with otitis media with effusion. *Otology & neurotology : official publication of the American Otological Society, American Neurotology Society [and] European Academy of Otology and Neurotology* **27**, 1089-1093 (2006).

21. Parkinson N, *et al.* Mutation at the Evi1 locus in Junbo mice causes susceptibility to otitis media. *PLoS genetics* **2**, e149 (2006).

22. Yeo SG, Park DC, Choo JH, Cha CI. Squamous metaplasia and BCL-6 in pediatric adenoid accompanied by otitis media with effusion. *Yonsei medical journal* **48**, 449-456 (2007).

23. Schachern PA, Cureoglu S, Tsuprun V, Paparella MM, Whitley CB. Age-related functional and histopathological changes of the ear in the MPS I mouse. *Int J Pediatr Otorhinolaryngol* **71**, 197-203 (2007).

24. Barzilai A, *et al.* Dynamics of interleukin-1 production in middle ear fluid during acute otitis media treated with antibiotics. *Infection* **27**, 173-176 (1999).

25. Smirnova MG, Birchall JP, Pearson JP. Evidence of T-helper cell 2 cytokine regulation of chronic otitis media with effusion. *Acta oto-laryngologica* **125**, 1043-1050 (2005).

26. Leichtle A*, et al.* TLR4-mediated induction of TLR2 signaling is critical in the pathogenesis and resolution of otitis media. *Innate immunity* **15**, 205-215 (2009).

27. Hornef N*, et al.* DNAH5 mutations are a common cause of primary ciliary dyskinesia with outer dynein arm defects. *American journal of respiratory and critical care medicine* **174**, 120-126 (2006).

28. Hilton JM*, et al.* Exome sequencing identifies a missense mutation in Isl1 associated with low penetrance otitis media in dearisch mice. *Genome biology* **12**, R90 (2011).

29. Patel JA*, et al.* Systemic cytokine response profiles associated with respiratory virus-induced acute otitis media. *The Pediatric infectious disease journal* **28**, 407-411 (2009).

30. Wiertsema SP*, et al.* Association of CD14 promoter polymorphism with otitis media and pneumococcal vaccine responses. *Clinical and vaccine immunology : CVI* **13**, 892-897 (2006).

31. Mao M*, et al.* The podosomal-adaptor protein SH3PXD2B is essential for normal postnatal development. *Mammalian genome : official journal of the International Mammalian Genome Society* **20**, 462-475 (2009).

32. Patel JA*, et al.* Association of proinflammatory cytokine gene polymorphisms with susceptibility to otitis media. *Pediatrics* **118**, 2273-2279 (2006).

33. Depreux FF*, et al.* Eya4-deficient mice are a model for heritable otitis media. *The Journal of clinical investigation* **118**, 651-658 (2008).

34. Eriksson PO, Li J, Ny T, Hellstrom S. Spontaneous development of otitis media in plasminogen-deficient mice. *International journal of medical microbiology : IJMM* **296**, 501-509 (2006).

35. Lucas JS*, et al.* Static respiratory cilia associated with mutations in Dnahc11/DNAH11: a mouse model of PCD. *Human mutation* **33**, 495-503 (2012).

36. Vogler C*, et al.* A novel model of murine mucopolysaccharidosis type VII due to an intracisternal a particle element transposition into the beta-glucuronidase gene: clinical and pathologic findings. *Pediatric research* **49**, 342-348 (2001).

37. Lee HY*, et al.* Antimicrobial activity of innate immune molecules against Streptococcus pneumoniae, Moraxella catarrhalis and nontypeable Haemophilus influenzae. *BMC infectious diseases* **4**, 12 (2004).

38. Li-Korotky HS, Swarts JD, Hebda PA, Doyle WJ. Cathepsin gene expression profile in rat acute pneumococcal otitis media. *The Laryngoscope* **114**, 1032-1036 (2004).

39. Koutnouyan HA, Baird A, Ryan AF. Acidic and basic FGF mRNA expression in the middle ear mucosa during experimental acute and chronic otitis media. *The Laryngoscope* **104**, 350-358 (1994).

40. Sautter NB, Delaney KL, Hausman FA, Trune DR. Tissue remodeling in the acute otitis media mouse model. *Int J Pediatr Otorhinolaryngol* **75**, 1368-1371 (2011).

41. Sale MM*, et al.* Evaluation of 15 functional candidate genes for association with chronic otitis media with effusion and/or recurrent otitis media (COME/ROM). *PLoS One* **6**, e22297 (2011).

42. MacArthur CJ, Pillers DA, Pang J, Degagne JM, Kempton JB, Trune DR. Gram-negative pathogen Klebsiella oxytoca is associated with spontaneous chronic otitis media in Toll-like receptor 4-deficient C3H/HeJ mice. *Acta oto-laryngologica* **128**, 132-138 (2008).

43. Born H, Peters A, Ettinger R. Inherited ADAMTS13 deficiency: unique presentation and treatment. *Pediatric blood & cancer* **50**, 956-957 (2008).

44. Nuytinck L, De Meester E, Van Thielen M, Govaerts P. Role of mannose-binding lectin (MBL2) genotyping in predicting the risk of recurrent otitis media (rOM). *Advances in experimental medicine and biology* **586**, 281-290 (2006).

45. Ramet M, Lofgren J, Alho OP, Hallman M. Surfactant protein-A gene locus associated with recurrent otitis media. *The Journal of pediatrics* **138**, 266-268 (2001).

46. Rivkin AZ, Palacios SD, Pak K, Bennett T, Ryan AF. The role of Fas-mediated apoptosis in otitis media: observations in the lpr/lpr mouse. *Hearing research* **207**, 110-116 (2005).

47. Kerschner JE, Meyer TK, Burrows A. Chinchilla middle ear epithelial mucin gene expression in response to inflammatory cytokines. *Archives of otolaryngology--head & neck surgery* **130**, 1163-1167 (2004).

48. Ubell ML, Khampang P, Kerschner JE. Mucin gene polymorphisms in otitis media patients. *The Laryngoscope* **120**, 132-138 (2010).

49. Santos-Cortez RL*, et al.* Rare A2ML1 variants confer susceptibility to otitis media. *Nature genetics* **47**, 917-920 (2015).

50. Gentile DA*, et al.* Cytokine gene polymorphisms moderate illness severity in infants with respiratory syncytial virus infection. *Human immunology* **64**, 338-344 (2003).

51. Kerschner JE*, et al.* A novel model of spontaneous otitis media with effusion (OME) in the Oxgr1 knock-out mouse. *Int J Pediatr Otorhinolaryngol* **77**, 79-84 (2013).

52. Bhutta MF, Hedge EA, Parker A, Cheeseman MT, Brown SD. Oto-endoscopy: a reliable and validated technique for phenotyping otitis media in the mouse. *Hearing research* **272**, 5-12 (2011).

53. Jang CH, Shin SH, Cho HH, Moon SJ, Cho YB. Expression of matrix metalloproteinase-9 and -2 in pediatric chronic otitis media with effusion. *Int J Pediatr Otorhinolaryngol* **70**, 1155-1158 (2006).

54. Humbert PO*, et al.* E2F4 is essential for normal erythrocyte maturation and neonatal viability. *Molecular cell* **6**, 281-291 (2000).

55. Leichtle A*, et al.* CC chemokine ligand 3 overcomes the bacteriocidal and phagocytic defect of macrophages and hastens recovery from experimental otitis media in TNF-/- mice. *Journal of immunology* **184**, 3087-3097 (2010).

56. Noben-Trauth K, Latoche JR. Ectopic mineralization in the middle ear and chronic otitis media with effusion caused by RPL38 deficiency in the Tail-short (Ts) mouse. *The Journal of biological chemistry* **286**, 3079-3093 (2011).

57. Rye MS*, et al.* FBXO11, a regulator of the TGFbeta pathway, is associated with severe otitis media in Western Australian children. *Genes and immunity* **12**, 352-359 (2011).

58. Leichtle A, Hernandez M, Pak K, Webster NJ, Wasserman SI, Ryan AF. The toll-Like receptor adaptor TRIF contributes to otitis media pathogenesis and recovery. *BMC immunology* **10**, 45 (2009).

59. Patel A, Gentile DA, Koehrsen J, Skoner DP. Association Between TGF-B1 Genotype and the Development of Otitis Media (OM) in Young Children during Respiratory Virus Season. *J Allergy Clin Immunol* **117**, (2006).

60. Wan H*, et al.* Foxa2 regulates alveolarization and goblet cell hyperplasia. *Development* **131**, 953-964 (2004).

61. Warren M*, et al.* A Sall4 mutant mouse model useful for studying the role of Sall4 in early embryonic development and organogenesis. *Genesis* **45**, 51-58 (2007).

62. Liao J*, et al.* Full spectrum of malformations in velo-cardio-facial syndrome/DiGeorge syndrome mouse models by altering Tbx1 dosage. *Human molecular genetics* **13**, 1577-1585 (2004).

63. Giovannini M*, et al.* Conditional biallelic Nf2 mutation in the mouse promotes manifestations of human neurofibromatosis type 2. *Genes & development* **14**, 1617-1630 (2000).

64. Voronina VA*, et al.* Inactivation of Chibby affects function of motile airway cilia. *The Journal of cell biology* **185**, 225-233 (2009).

65. Thut CJ, Rountree RB, Hwa M, Kingsley DM. A large-scale in situ screen provides molecular evidence for the induction of eye anterior segment structures by the developing lens. *Developmental biology* **231**, 63-76 (2001).

66. Diez-Roux G*, et al.* A high-resolution anatomical atlas of the transcriptome in the mouse embryo. *PLoS biology* **9**, e1000582 (2011).

67. Ward LD, Kellis M. HaploReg: a resource for exploring chromatin states, conservation, and regulatory motif alterations within sets of genetically linked variants. *Nucleic Acids Res* **40**, D930-934 (2012).

68. ENCODE Project Consortium. An integrated encyclopedia of DNA elements in the human genome. *Nature* **489**, 57-74 (2012).

69. Wu C*, et al.* BioGPS: an extensible and customizable portal for querying and organizing gene annotation resources. *Genome biology* **10**, R130 (2009).

70. Bastian F, Parmentier G, Roux J, Moretti S, Laudet V, Robinson-Rechavi M. Bgee: Integrating and Comparing Heterogeneous Transcriptome Data Among Species. *Data Integration in the Life Sciences* **5109**, 124-131 (2008).
